# Early Magnetic Resonance Detection of Natalizumab-Related Progressive Multifocal Leukoencephalopathy in a Patient with Multiple Sclerosis

**DOI:** 10.1155/2013/415873

**Published:** 2013-03-10

**Authors:** Guglielmo Manenti, Simone Altobelli, Marco Nezzo, Marco Antonicoli, Erald Vasili, Luca Neroni, Roberto Floris, Giovanni Simonetti

**Affiliations:** Department of Diagnostic and Molecular Imaging, Interventional Radiology and Radiation Therapy, Fondazione Policlinico “Tor Vergata”, Viale Oxford 81, 00133 Rome, Italy

## Abstract

Diagnosis of progressive multifocal leukoencephalopathy is usually based on the clinical presentation, on the demonstration of the brain lesions at the magnetic resonance imaging examination, and on the detection of the JC virus DNA in the cerebrospinal fluid with high sensitive polymerase chain reaction. The role of magnetic resonance imaging specifically in natalizumab-associated progressive multifocal leukoencephalopathy is strengthening, and it is gaining importance not only as an irreplaceable diagnostic tool but also as a surveillance and risk stratifying tool in treated patients. While other imaging techniques such as computed tomography lack sensitivity and specificity, magnetic resonance performed with morphological and functional sequences offers clinicians the possibility to early identify the stage of the disease and the emergence of an immune reconstitution inflammatory syndrome after natalizumab blood removal plasmapheresis.

## 1. Case Report

**Figure 1:**
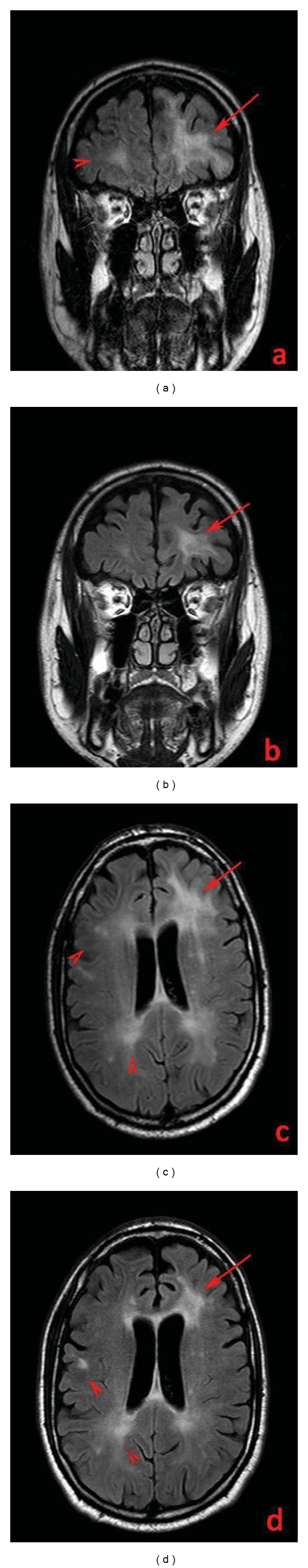
T2w FLAIR images (Philips Achieva 3T, TR = 9000 ms; TE = 123 ms; inversion time TI = 2500 ms; FA 180). Juxtaventricular peri- and paratrigonal white matter lesion underneath the frontal lobe of the left hemisphere (arrows) in a 44-year-old man with a 12-year history of MS and affected by natalizumab-related PML in the coronal (a), (b) and axial plan (c), (d). Lesion reduction between December 2011 (a), (c) and May 2012 (b), (d). A bilateral white matter periventricular lesion load related to MS is also seen (arrow heads).

**Figure 2:**
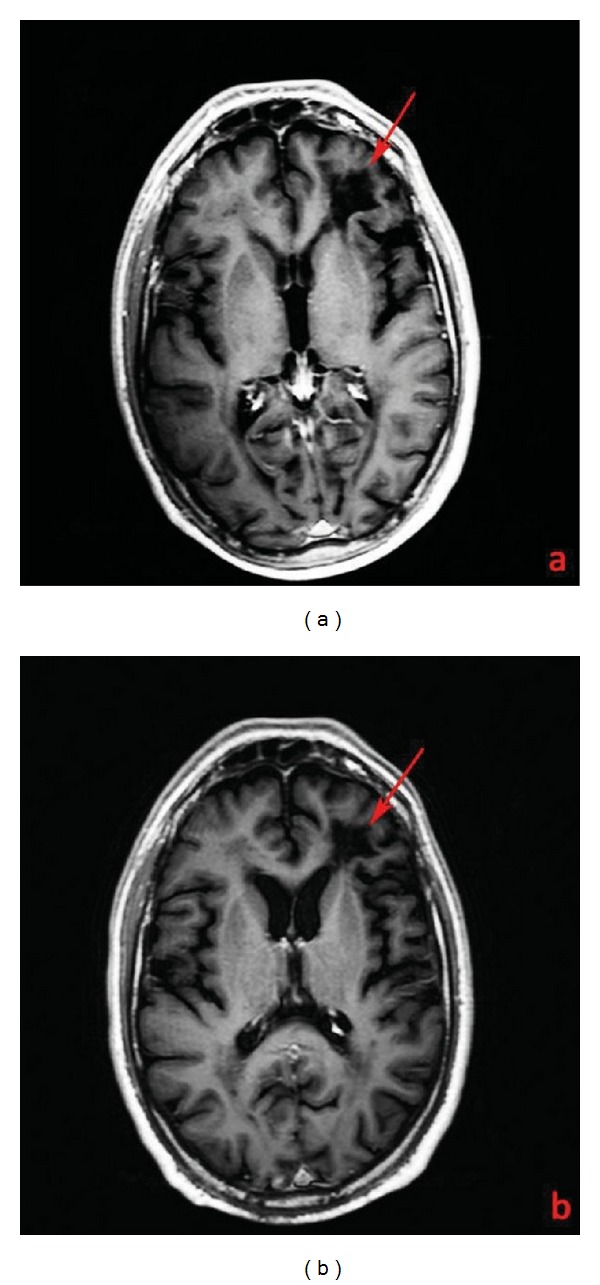
T1 post-Gd images (Philips Achieva 3T, TR/TE = 500 msec./14 msec, 15 cc Gadobenate Dimeglumine, Multihance) in December 2011 (a) and May 2012 (b) of the white matter lesion underneath the frontal lobe of the left hemisphere (arrows) without detectable contrast enhancement in a 44-year-old man with a 12-year history of MS affected by natalizumab-related PML.

**Figure 3:**
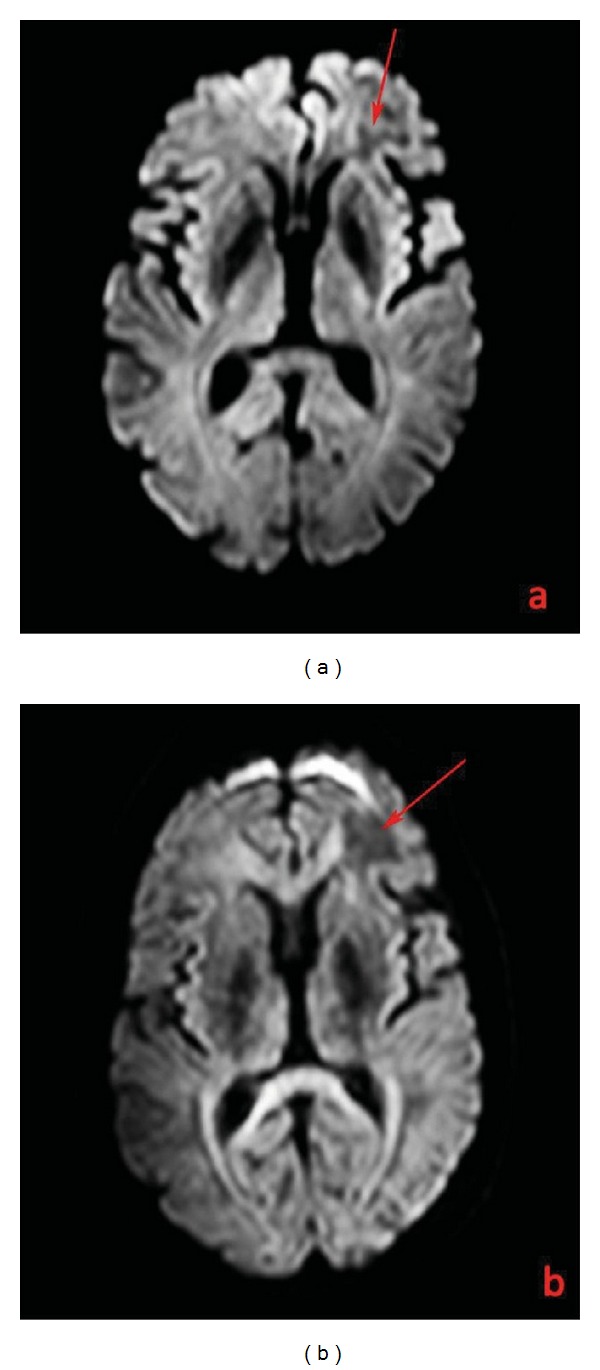
DWI ADC map (Philips Achieva 3T, TR = 4500 ms; TE = 112 ms) in December 2011 (a) and May 2012 (b) of the white matter lesion underneath the frontal lobe of the left hemisphere (arrows) with some diffusion restriction in a 44-year-old man with a 12-year history of MS affected by natalizumab-related PML.

**Figure 4:**
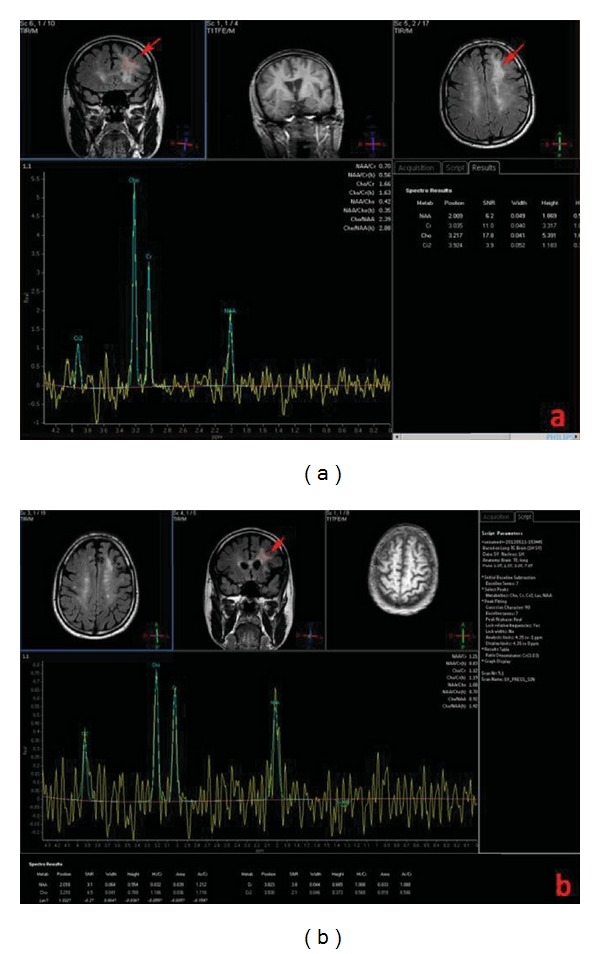
MRS (Philips Achieva 3T, TR/TE = 2000/40, increased choline, inverted Cho/Cr ratio, decreased N-acetyl-aspartate between December 2011 (a) and May 2012 (b) of the white matter lesion underneath the frontal lobe of the left hemisphere (arrows) at MRS in a 44-year-old man with a 12-year history of MS affected by natalizumab-related PML. May 2012 MRS showed a decrease in Cho/Cr ratio and increased N-acetylaspartate.

A 44-year-old man with a 12-year-history of multiple Sclerosis (MS) was treated with interferon therapy till June 2009 when it was decided to introduce natalizumab (Tysabri; Biogen Idec, Weston, MA, USA) due to symptomatology worsening (reduction in walking autonomy) which quickly regressed after three months of therapy. In December 2011, the patient reported a memory and concentration deficit and presented several episodes of verbal aggression, mood changes, and suicidal thoughts. The patient was admitted at the Neurology Department of our Institution for further clinical and radiological examinations. A magnetic resonance (MR) scan on a 3T scanner (Philips Achieva 3T) was performed using the following sequences: T1 and T2 weighted, fluid attenuated inversion recovery (FLAIR), diffusion weighted imaging (DWI), T1 post-gadolinium (Gd), and single- and multivoxel point resolved spectroscopy (PRESS). The MR exam showed a wide subcortical lesion with an irregular shape which involved the semioval center underneath the frontal lobe of the left hemisphere with a minor involvement of subcortical white matter of the right hemisphere and of the anterior commissure (Figure 1). The lesion showed a high signal intensity on T2-weighted and FLAIR images, low signal on T1-weighted images, no contrast-enhancement (CE) after endovenous gadolinium administration (Figure 2), no restricted diffusion (Figure 3), elevated lactate and myoinositol levels, and an increase of choline with a reduction of creatine (reversed ratio) and NAA (N-acetyl-aspartate) in both monovoxel and multivoxel spectroscopies (Figure 4). Due to magnetic resonance imaging (MRI) findings, it was decided by the clinicians to abrupt Natalizumab therapy and perform the spinal tap. A low JCV DNA burden was found in the cerebrospinal fluid (CSF) with high sensitive polymerase chain reaction (PCR). Subsequently, it was decided to start plasmapheresis (PLEX) treatment to obtain the complete drug removal. At the followup with MR, in February 2012, no signs of IRIS (immune reconstitution inflammatory syndrome) were identified in agreement with the regression of the symptomatology. In May 2012, another MR examination was performed at 5 months from the abruption of the therapy which showed the slight reduction of the lesion extension, of choline and lactate levels, and no CE after Gd administration.

## 2. Discussion

Progressive multifocal leukoencephalopathy (PML) is caused by JC virus (JCV), identified in 1971, which is a nonenveloped, double-stranded DNA virus (5,1 kilobases) in the family of Polyomaviridae. Its DNA codes for six nonstructural proteins, three capsid proteins (VP1, VP2, VP3), and contains a noncoding region which acts as a transcription regulatory region that may affect the viral cellular tropism [1–4]. The PML neuropathology was firstly reported in 1958 when was performed a brain tissue examination from two cases of chronic lymphocytic leukemia and one case of Hodgkin lymphoma, although the classic triad of symptoms (visual deficits, motor weakness, and cognitive impairment) was previously observed [5]. Actually, the PML is mostly observed in patients affected by acquired immune deficiency syndrome (AIDS) and transplanted patients, while it is less documented in immunoproliferative disorders. Recently it has occasionally been observed in patients treated with biologic drugs such as natalizumab (Tysabri) which is an alpha-4-beta-1 integrin inhibitor approved for the treatment of relapsing remitting MS and Efalizumab which is an immunosuppressive recombinant-humanized IgG1 monoclonal antibody that binds to human CD11a, used in psoriasis therapy. JCV has an ubiquitous distribution from 50% to 60% of adults aged from 20 to 50 years demonstrating antibodies. MS is an autoimmune disease which causes demyelination, axonal loss, and neurodegeneration and leads to progressive neurological disability. Without treatment, the disability at 15/20 years after the disease onset is estimated as 50% [6]. Natalizumab is a humanized IgG4 monoclonal antibody that binds the subunit a4 of very late antigen-4 (VLA-4) on the surface of leucocytes. This molecule is part of the large family of the integrins which mediate the adhesion of cells with the extracellular matrix. In the MS, natalizumab inhibits the adhesion of leucocytes to the endothelial cells of the brain vessels, preventing migration in the extracellular space of the central nervous system (CNS) without interfering with leukocytes proliferation and activation [7–11]. It was approved by the Food and Drug Administration (FDA) for the treatment of remitting relapsing MS in November 2004 on the basis of the AFFIRM (natalizumab safety and efficacy in relapsing remitting multiple sclerosis), SENTINEL (safety and efficacy of natalizumab in combination With remicade in the treatment of Crohn's disease), and other studies which also showed a significant improvement in health related quality of life (HRQoL), abrupted in February of 2005 according to the identification of the first three cases of PML and restored in June 2006 as part of a restricted distribution program (TOUCH: Tysabri Outreach Unified Commitment to Health) [12–14]. Till June 2011, 88100 patients had been dosed with natalizumab, and the risk of PML is estimated to be 1,66 in 1000 patients and depends on the therapy duration and number of doses administered. Till August 2011, there were 150 confirmed cases of PML, 85 of which in Europe. There is no animal model for JC virus infection, but its replication was demonstrated in astrocytes, oligodendrocytes, uroepithelial cells, CD34+ cells, CD19 cells, and cerebellar granular cells [15–17]. CNS infection leads to the development of multiple or solitaire demyelinating lesions of the white matter underneath the parietooccipital lobes, at the corpus callosum, and basal ganglia [18, 19]. Histopathologically, PML is characterized by extensive and progressive demyelination with absence of the inflammatory response which permits the differentiation of this pathology from other demyelination diseases such as MS. The way of transmission is still uncertain, but recent acknowledgments identify the tonsils as the primary site of infection from which JC virus, after a primary replication, may reach the blood circulation and the bone marrow [20, 21]. Latent infection has an high prevalence (33–60%) in healthy adult population and the virus persists inoffensive in the bone marrow and kidney affecting CD-34 cells, B lymphocytes progenitors, and uroepithelial cells. Immunosuppression and lack in CD8+ immunitary response, along with the natalizumab-associated increase of peripheral blood levels of CD-34 and B lymphocytes progenitors that act like virus carriers and the increase in their nucleus levels of transcription factors such as SPI-B which promotes viral DNA replication, seems to be related to the colonization of the CNS and the development of PML [22]. Natalizumab-related PML shows unclear gender and age predilection. Diagnosis of PML in patient undergoing natalizumab therapy resides in an accurate clinical examination which can demonstrate the typical triad of symptoms (cognitive impairment, visual deficit, and motor dysfunction), MR examination and laboratory results which can confirm the diagnosis by the identification of JC virus DNA in CSF or in the brain tissue with PCR [23]. Neuroimaging today is an irreplaceable tool for diagnosis. Computed tomography (CT) lacks sensitivity and specificity demonstrating in PML patients, only nonspecific hypodensities localized in the white matter and sometimes (<10% of cases) a CE of the lesion [24, 25]. At MRI, PML is characterized by single- (majority of cases) or multifocal oval white matter lesions. With disease progression, the lesions may become confluent or grow as a giant white matter plaque. These lesions are hypointense on T1- and hyperintense on T2-weighted images, compared to normal white matter. Common localizations are the parietal and occipital lobes and the corpus callosum while cerebellum, and brainstem involvement is occasionally seen. Usually due to the lack of inflammatory response, no CE is demonstrable but sometimes it is present as a faint peripheral ring. In the majority of cases, there is no mass effect on the contiguous structures [26]. Lesion signal on DWI depends on the age and activity of the lesion: in early ones, diffusion could appear restricted at the borders, while in older it could be increased in the center. Higher B values (>2000) can better depicts lesion borders. PML localizations show abnormal fractional anisotropy values ensuring an earlier identification of the pathology extension on diffusion tensor imaging (DTI). On magnetic resonance spectroscopy (MRS) lesions are characterized by an increased choline, elevated lactate, variable myoinositol, and decrease of N-acetyl-aspartate, although spectra obtained can differ from the center to the border of the lesion. Positron emission tomography/computed tomography (PET/CT) in these patients shows hypometabolic lesions in the majority of cases and results useful in differentiating them from lymphomas. Despite pathology appearance in the different imaging modalities, MRI findings seem to be the most specific, and this imaging technique is going to play a central role not only in the early and sometimes presymptomatic disease diagnosis, but also in the assessment of treatment response and prognosis [27–29]. It is not always is possible to obtain a positive result at CSF q-PCR in patient with strong suspicion of PML at clinical and MRI examination. In our case the JCV DNA identification required a highly sensitive PCR as the viral load was low (<500 DNA copies/tl). PML differential diagnosis includes (Table 1) primary CNS lymphoma, ischemic infarct, acute disseminated encephalomyelitis (ADEM), epstein-barr virus- (EBV-) induced encephalitis, toxoplasmosis, early stage brain abscess, and MS relapse. Primary CNS lymphoma at CT typically appears as a high-density lesion in a central hemispheric location that often reaches or crosses the midline, characterized at MRI by intermediate-to-low signal intensity on T1-weighted images, and either isointense or hypointense signal on T2-weighted images, restricted diffusion, typical homogeneus CE and elevated lipid peaks and Cho/Cr (choline/creatine) ratio on MRS. Ischemic infarct appears at CT as a low-density lesion occupying a vascular territory with some swelling and at MRI as an area of hyperintensity on FLAIR sequences after 6 hours from onset, abnormally perfused on PWI (perfusion weighted imaging) and with increased lactate and decreased NAA on MRS. ADEM may appear at CT as scattered low-density areas and at MRI as multiple areas of hyperintensity at the gray-white matter junction demonstrating variable restricted diffusion and a possible ring enhancement. CT in EBV induced encephalitis may document low-density parenchymal lesions characterized at MRI by restricted diffusion and increased myoinositol and choline. Toxoplasmosis is characterized by no pathognomonic lesions at CT, hypointense and hyperintense areas, respectively, on T1- and T2-weighted images with ring enhancement, edema, elevated lactate, and lipids at MRI. Early-stage brain abscess appearance at CT consists in a lesion with a central low-density core with peripheral iso-hyperdense ring that is enhanced after contrast medium administration and at MRI in a lesion with a hypointense core and peripheral low-intensity area (edema) on T1-weighted vice versa on T2-weighted and FLAIR images, characterized by restricted diffusion and elevated succinate and acetate on MRS. MS relapse is characterized at CT by white matter hypodensities abutting ventricles and at MRI by high signal lesions on T2-weighted and FLAIR images that are enhanced when active on T1-weighted images after Gd administration, with possible involvement of brainstem and Cerebellum and with decreased NAA on MRS. Currently there are no specific antiviral agents to treat PML, although recent findings have shown that Mirtazapine and a lipid ester of Cidofovir (CMX001) may inhibit, respectively, JCV entry and replication in glial cells [30]. The mainstay of PML treatment is immune reconstitution which is obtainable with HAART (highly active antiretroviral therapy) in AIDS related PML or with the drug removal in natalizumab associated disease. Desaturation of the integrin receptors occurs when serum drug level is less than 1 *μ*g/*μ*L. PLEX is needed and strongly recommended in patients that develop PML during natalizumab treatment to accelerate its blood removal. Patients undergoing PLEX should be monitored with clinical and radiological examination to asses PML regression or to early identify the onset of an IRIS. PML prognosis reflects lesions' localization, the ability to achieve an early diagnosis, and the immune reconstitution.

## Figures and Tables

**Table 1:** Differential diagnosis of PML.

	CT	MRT
PML	(i) Nonspecific hypodensities localized in the white matter	(i) Hypointense in T1 and hyperintense in T2, single lesion, round or oval, (majority of cases) lesion or multifocal white matter lesions (ii) Signal at DWI sequences depends on the age and activity of the lesion but is often restricted (iii) Abnormal fractional anisotropy values on DTI (iv) At MRS, lesions are characterized by an increased choline, elevated lactate, variable myoinositol, decrease of N-acetylaspartate, and increased choline/creatine ratio

CNS lymphoma	(i) CT typically shows a high-density (70%) lesion in a central hemispheric location, which often reaches or crosses the midline (ii) Intense and homogeneous CE is the hallmark of primary CNS lymphoma	(i) Lesion appears at MRI with intermediate-to-low signal intensity on T1-weighted images and either isointense or hypointense signal on T2-weighted images (ii) Diffusion is often restricted (iii) Elevated lipid peaks and high Cho/Cr ratios on MRS (iv) The intense homogeneous enhancement is the hallmark of primary CNS lymphoma

Ischaemic infarct	(i) Usually CT demonstrates a low-density lesion occupying a vascular territory with some swelling	(i) Hyperintense on DWI scans and hypointense on ADC maps (ii) No signs on FLAIR in the first 6 h from onset, with areas of hyperintensity evolving thereafter (iii) Regions of brain tissue that are abnormally perfused on PWI (iv) NAA decrease and Lactate increase on MRS (v) Stenoses, occlusions, and dissections on MRA

ADEM	(i) CT scan is relatively insensitive, but may show scattered low-density areas	(i) T2W and FLAIR images usually show multiple regions of hyperintensity at the gray-white junction, in the brainstem, cerebellum, and basal ganglia (ii) Solid or ring enhancement can be seen (iii) There can be variable diffusion restriction (iv) Spectroscopy can show low NAA

EBV-induced encephalitis	(i) CT results may be negative (ii) Low-density parenchymal lesions (iii) Brain atrophy	(i) MRI results may be negative (ii) Restricted diffusion on DWI (iii) Presence of an increase in myoinositol together with choline values on MRS

Toxoplasmosis	(i) CT appearance of Toxoplasma encephalitis is not pathognomonic (ii) Ring enhancing may be present on contrast-enhanced CT	(i) On T1-weighted MRI, the lesions are hypointense relative to brain tissue (ii) On T2-weighted MRI, foci of infections are usually hyperintense (iii) Ring enhancing may be present after Gd administration (iv) Active lesions are often surrounded by edema (v) Elevated lactate and lipid

Early stage brain abscess	(i) Central low-density core (ii) Iso-hyperdense ring (iii) Peripheral low density (edema) (iv) Ring enhancement	(i) T1: central low intensity, peripheral low intensity (vasogenic edema), ring enhancement after Gd administration (ii) T2/FLAIR: central high intensity, peripheral high intensity (vasogenic oedema) (iii) DWI/ADC: high DWI signal is usually present (iv) Elevation of succinate and acetate is relatively specific

MS relapse	(i) Atrophy (ii) Periventricular or elsewhere white matter hypodensities	(i) High signal on T2-weighted and FLAIR MRI sequences (ii) When actively inflamed, often enhanced with gadolinium contrast
	(iii) Areas of abnormal enhancement	(iii) Position abutting ventricles (often perpendicular) (iv) Juxtacortical position (gray-white junction) (v) Involvement of brainstem, cerebellum, or corpus callosum (vi) Decrease in NAA and creatine, and increase of choline on MRS

## Abbreviations

MR: Magnetic resonance
MS: Multiple sclerosis
FLAIR: Fluid attenuated inversion recovery
DWI: Diffusion weighted imaging
Gd: Gadolinium
PRESS: Point resolved spectroscopy
CE: Contrast enhancement
NAA: N-acetylaspartate
CSF: Cerebrospinal fluid
MRI: Magnetic resonance imaging
PCR: Polymerase chain reaction
PLEX: Plasmapheresis
IRIS: Immune reconstitution inflammatory syndrome
PML: Progressive multifocal leukoencephalopathy
JCV: JC virus
AIDS: Acquired immune deficiency syndrome
VLA-4: Very late antigen-4
CNS: Central nervous system
FDA: Food and drug administration
AFFIRM: Natalizumab safety and efficacy in relapsing remitting multiple sclerosis
SENTINEL: Safety and efficacy of natalizumab in combination with remicade in the treatment of Crohn's disease
HRQoL: health-related quality of life
TOUCH: Tysabri outreach Unified Commitment to Health
CT: Computed tomography
DTI: Diffusion tensor imaging
MRS: Magnetic resonance spectroscopy
PET/CT: Positron emission tomography/computed tomography
ADEM: Acute disseminated encephalomyelitis
EBV: Epstein-Barr virus
PWI: Perfusion-weighted imaging
HAART: Highly active antiretroviral therapy
Cho/Cr: Choline/creatine.
